# Systematic Selection of Age-Associated mRNA Markers and the Development of Predicted Models for Forensic Age Inference by Three Machine Learning Methods

**DOI:** 10.3389/fgene.2022.924408

**Published:** 2022-07-01

**Authors:** Xiaoye Jin, Zheng Ren, Hongling Zhang, Qiyan Wang, Yubo Liu, Jingyan Ji, Jiang Huang

**Affiliations:** Department of Forensic Medicine, Guizhou Medical University, Guiyang, China

**Keywords:** mRNA, machine learning, forensic age estimation, genetic markers, aging

## Abstract

Aging is usually accompanied by the decline of physiological function and dysfunction of cellular processes. Genetic markers related to aging not only reveal the biological mechanism of aging but also provide age information in forensic research. In this study, we aimed to screen age-associated mRNAs based on the previously reported genome-wide expression data. In addition, predicted models for age estimations were built by three machine learning methods. We identified 283 differentially expressed mRNAs between two groups with different age ranges. Nine mRNAs out of 283 mRNAs showed different expression patterns between smokers and non-smokers and were eliminated from the following analysis. Age-associated mRNAs were further screened from the remaining mRNAs by the cross-validation error analysis of random forest. Finally, 14 mRNAs were chosen to build the model for age predictions. These 14 mRNAs showed relatively high correlations with age. Furthermore, we found that random forest showed the optimal performance for age prediction in comparison to the generalized linear model and support vector machine. To sum up, the 14 age-associated mRNAs identified in this study could be viewed as valuable markers for age estimations and studying the aging process.

## Introduction

Aging is a normal phenomenon and the most complicated biological process in nature. Research on the biological mechanisms of aging can contribute to understanding the pathogenesis of age-associated diseases like Alzheimer’s disease and Parkinson’s disease (Gomez-Verjan et al., 2018). In forensic research, age prediction can provide informative investigative clues, especially for some trace samples like blood stain, saliva, and seminal stain. Furthermore, age is also viewed as an important index for sentencing young criminals in legal cases. A previous study pointed out that there were some featured imprints related to the body physiological function during the aging process (López-Otín et al., 2013). Accordingly, screening age-associated molecular markers is significant to understanding the aging process and forensic practice.

In forensic science, researchers commonly infer age information of unknown samples found in crime scenes by morphological methods (Kotěrová et al., 2018; Meng et al., 2019; Gok et al., 2020). For example, Meng et al. estimated age by the color change of the costal cartilage; Gok et al. utilized two methods (dental pulp visibility and tooth coronal index) to infer age information; Koterova et al. conducted age estimation by the changes of the pubic symphysis and the auricular surface of the hip bone. However, these morphological methods possessed relatively low prediction accuracy; Therefore, they were prone to the influence of the subjective. Therefore, it is necessary to select novel genetic markers for age estimation. In recent years, with the development of aging research, biological processes associated with aging have been identified, like mitochondrial dysfunction, genomic instability, and epigenetic changes (López-Otín et al., 2013; Kennedy et al., 2014). Molecular markers related to these biological processes were selected for age prediction (Kennedy et al., 2013; Ibrahim et al., 2016; Demanelis et al., 2021). Nonetheless, it is of note that these methods still possess relatively low accuracy for age estimation. DNA methylation, one of the epigenetic changes, has shown to be an ideal biomarker for age prediction. Until now, a host of age-associated DNA methylation markers have been selected, and various prediction models have been built (Naue et al., 2017; Feng et al., 2018). Nonetheless, the quantification method of DNA methylation needs to conduct the bisulfite conversion procedure, which may lead to DNA fragmentation or DNA damage. Accordingly, these methods adversely detected forensic trace samples, which limited their application in forensic practices. Interestingly, previous studies have found that gene expression levels showed high correlations with aging (Pan et al., 2007; de Magalhães et al., 2009; Peters et al., 2015; Huan et al., 2018; Mamoshina et al., 2018). Forensic researchers also selected age-associated mRNA and miRNA markers for forensic age estimation (Nakamura et al., 2012; Zubakov et al., 2016; Deng et al., 2017; Fang et al., 2020; Wang et al., 2022). Even so, it is essential to screen more markers associated with age, which is beneficial to inferring the biological age of unknown individuals better.

In this study, we re-analyzed the previously reported genome-wide expression data (Votavova et al., 2011) and screened mRNAs related to aging. Next, these initially selected mRNA makers were further screened by the machine learning method (random forest, RF). Finally, we compared the performances of three machine learning methods for age estimations based on screened mRNA markers.

## Materials and Methods

### Sample Information

Votavova et al. (2011) assessed the effect of smoking on maternal cells at the transcription level. In the study, they collected blood samples of 52 nonsmokers and 20 smokers whose ages ranged from 18 to 41. However, only 46 nonsmokers and 19 smokers were engaged for the following analysis. Sample information used in this study is given in Supplementary Table S1. Expression levels of 24,526 transcripts in these samples were detected by the HumanRef-8 v3 Expression BeadChips (Illumina, San Diego, CA, United States). Based on the data (GSE27272), we aimed to screen age-associated mRNAs. Detailed experimental procedures were reported in the study (Votavova et al., 2011). In brief, RNA samples were extracted and purified from blood samples by using the LeukoLOCK™ Total RNA Isolation System (Ambion, Austin, TX, United States). Second, cRNA was synthesized and biotinylated by using the llumina TotalPrep RNA amplification kit (Ambion). The hybridization reaction of each cRNA sample was conducted on the beadchips and scanned by using the BeadArray Reader. Finally, the obtained raw data were processed and normalized by the quantile method in the Lumi package of R software (www.r-project.org).

### Selection of Age-Associated mRNAs

First, all samples (46 nonsmokers and 19 smokers) were classified into two groups to select age-associated mRNA markers; one group included individuals whose ages were from 18 to 30 and was viewed as the younger group; the other group included the remaining individuals and was treated as the older group. Expression level comparisons between two groups were conducted by the GEO2R online tool. Differentially expressed genes were identified when they had *p* values < 0.05 and |logFC| > 0.35 (Votavova et al., 2011). Next, these initially selected mRNA markers were further screened according to their expression patterns between smokers and nonsmokers. The transcripts that showed significantly different expression patterns between smokers and nonsmokers were excluded from the following study. Third, the remaining mRNA markers were further assessed by the RF method of R software to evaluate their importance in age prediction. In a nutshell, 10 fold cross-validation was used to evaluate the performance of models by sequentially reducing the number of mRNA markers, according to their importance index. The aforementioned procedure was repeated 10 times. Next, the optimal number of mRNA markers for age prediction was determined by comparing the cross-validation error of each model built with different numbers of mRNA markers. Spearman correlation coefficients between selected mRNA markers and different ages were estimated and visually shown by the Sangerbox 3.0 online tool (http://vip.sangerbox.com/home.html). Models for age predictions were built by the generalized linear model (GLM), RF, and support vector machine (SVM) using all samples. For SVM, we used the tune function of the e1071 v1.7-3 package to optimize the SVM model and then employed the best parameters to build the SVM model with the kernel of linear. For GLM and RF, they were built by the stats v3.6.1 and randomForest v4.6-14 for age estimation based on the default configuration. The performance of different models was compared by two indexes: root mean squared error (RMSE) and mean absolute error (MAE). The formulae of RMSE and MAE are listed as follows:

RMSE = sqrt(mean((pred - obs)^2
MAE = mean(abs(pred-obs))

Note: pred and obs indicate predicted and actual results.

The gene set enrichment analysis of screened genes related to age was conducted by the clusterProfiler v3.14.3 of R software. Background genes were chosen from the Molecular Signatures Database (Liberzon et al., 2011) and the KEGG rest API (https://www.kegg.jp/kegg/rest/keggapi.html). We used the Benjamini–Hochberg method to correct the statistical significance of inputted gene sets.

## Results and Discussion

### Selection of Age-Associated mRNAs

First, 65 samples were classified into two groups with different age ranges to select differentially expressed mRNAs between the two groups. According to the criteria mentioned earlier, 283 mRNAs were selected from the whole genome transcript level (Supplementary Table S2). Since smoking may affect the expression levels of different genes, we also assessed differentially expressed mRNAs between smokers and non-smokers. Results revealed that 315 mRNAs displayed different expression levels between smokers and non-smokers (Supplementary Table S3). Therefore, nine overlapped mRNAs between two sets of differentially expressed mRNAs were eliminated from the following analysis to avoid the negative effect of smoking on the age prediction. Finally, 274 candidate mRNA markers related to age were employed for further analysis.

Based on the selected 274 mRNA markers, we used RM to further screen age-related mRNAs. First, we assessed the importance index (mean decrease in node impurity) of these mRNA markers in age estimation (Supplementary Table S4). The mean decrease in node impurity can measure the effect of each variable on the impurity of predicted results; it is calculated by the residual sum of squares for regression analysis. The larger the mean decrease in node impurity of the variable is, the more important the variable is. To determine the optimal number of mRNA markers, the cross-validation error of each model built with different number of mRNA makers was assessed, as shown in Supplementary Figure S1. We found that a significant decrease in the cross-validation error was observed when the number of mRNA makers was 14. Even though the lowest cross-validation error was observed by using more mRNA markers, we selected the top 14 mRNA markers, according to the parsimony principle. The basic information of these 14 mRNAs is given in Supplementary Table S5.

**FIGURE 1:**
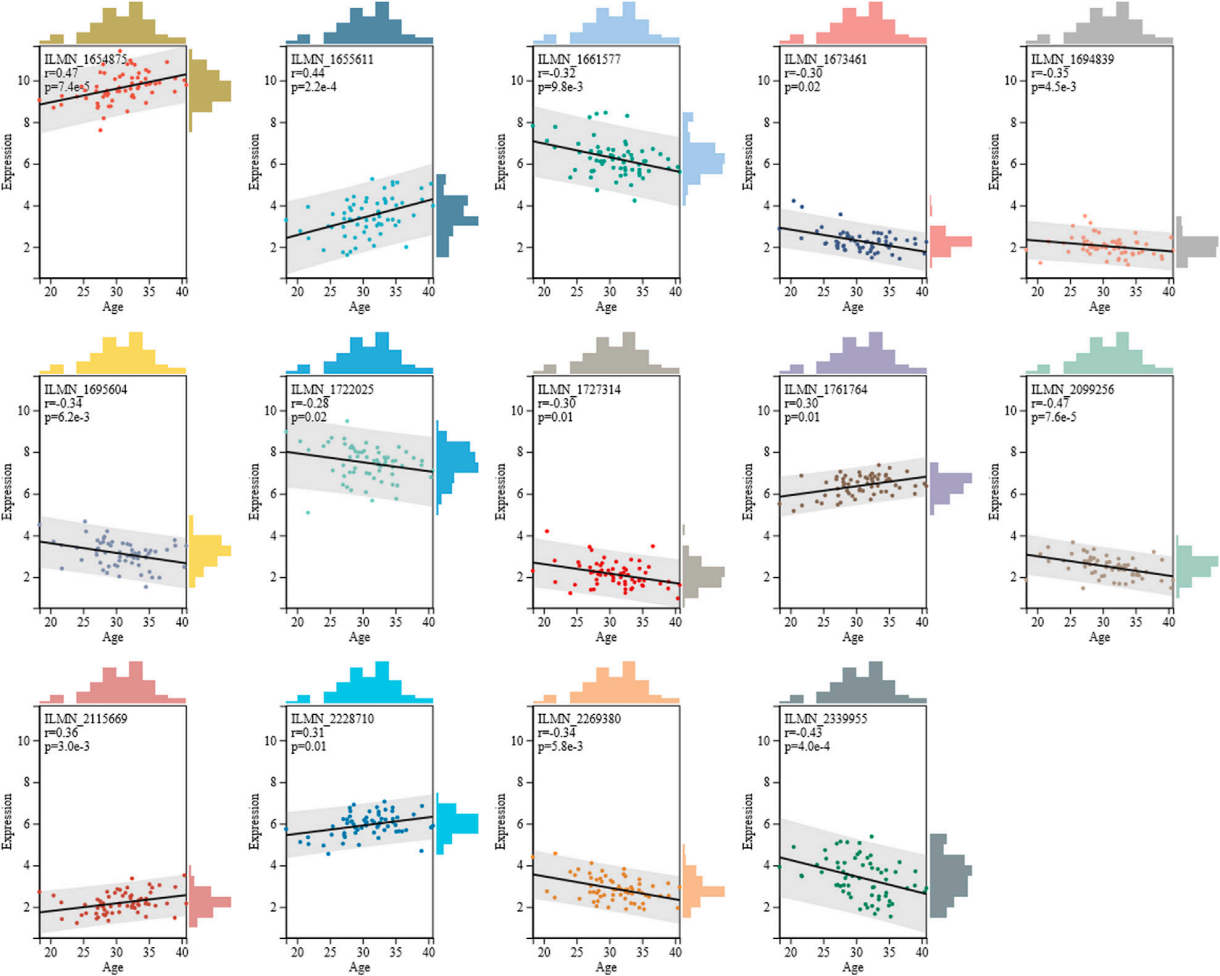
Spearman correlation coefficient of 14 mRNAs with age. Detailed information of 14 mRNAs is given in Supplementary Table S5.

Next, we assessed the correlation coefficient of 14 mRNAs with age, as shown in Figure 1. Results showed that five mRNAs exhibited positive correlation with age, and their correlation coefficient ranged from 0.30 to 0.47. Furthermore, nine mRNAs displayed negative correlation with age, and their correlation coefficient ranged from -0.47 to -0.28. Compared to six age-associated miRNAs selected by Fang et al. (2020), the 14 mRNAs presented in this study showed higher correlation with age, implying that these 14 mRNAs might be more beneficial for age estimations.

### Development of age Prediction Models by Three Machine Methods

Machine learning could build a high-efficient and accurate predicted model for various purposes, which has shown great promising aspects in clinical and forensic research (Obermeyer and Emanuel, 2016; Liu et al., 2020; Peña-Solórzano et al., 2020; Santolaria, 2021). For RF, it is an ensemble learning algorithm by developing a number of decision trees. Predicted results were determined by these decision trees. Accordingly, RF can avoid overfitting for the training data set and possess better performance than a decision tree. More importantly, RF can build a high-performance model for a variety of data sets with little configuration (https://www.stat.berkeley.edu/∼breiman/RandomForests/cc_home.htm). For SVM, it is one of the most robust machine learning methods. Compared to other machine learning methods, SVM is not prone to building an overfitting predicted model and shows high prediction accuracy for all kinds of data (Ghatak, 2017). For GLM, it is a simple learning method and is used to construct the predicted model to measure relationships between targeted variables and explanatory variables by the linear function. Given that selected mRNAs presented linear relationships with aging to some degree, we also explored the power of GLM for age estimation.

**FIGURE 2:**
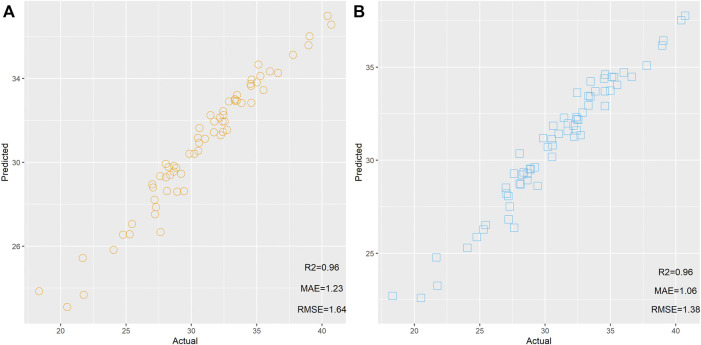
Scatter plot of the predicted age and actual age by random forest based on 274 **(A)** and 14 mRNAs **(B)**.

First, two RF models were built based on 274 and 14 mRNAs. These two models were used to predict the age of 65 samples, respectively. The scatter plot of the predicted age and actual age is shown in Figure 2. We found that the same *R* ^*2*^ between the predicted results and actual results could be observed from 274 and 14 mRNAs, but the model built based on 14 mRNAs showed lower MAE and RMSE than the model based on 274 mRNAs, implying that these 14 mRNAs showed better performance for age estimation than the 274 mRNAs.

**FIGURE 3:**
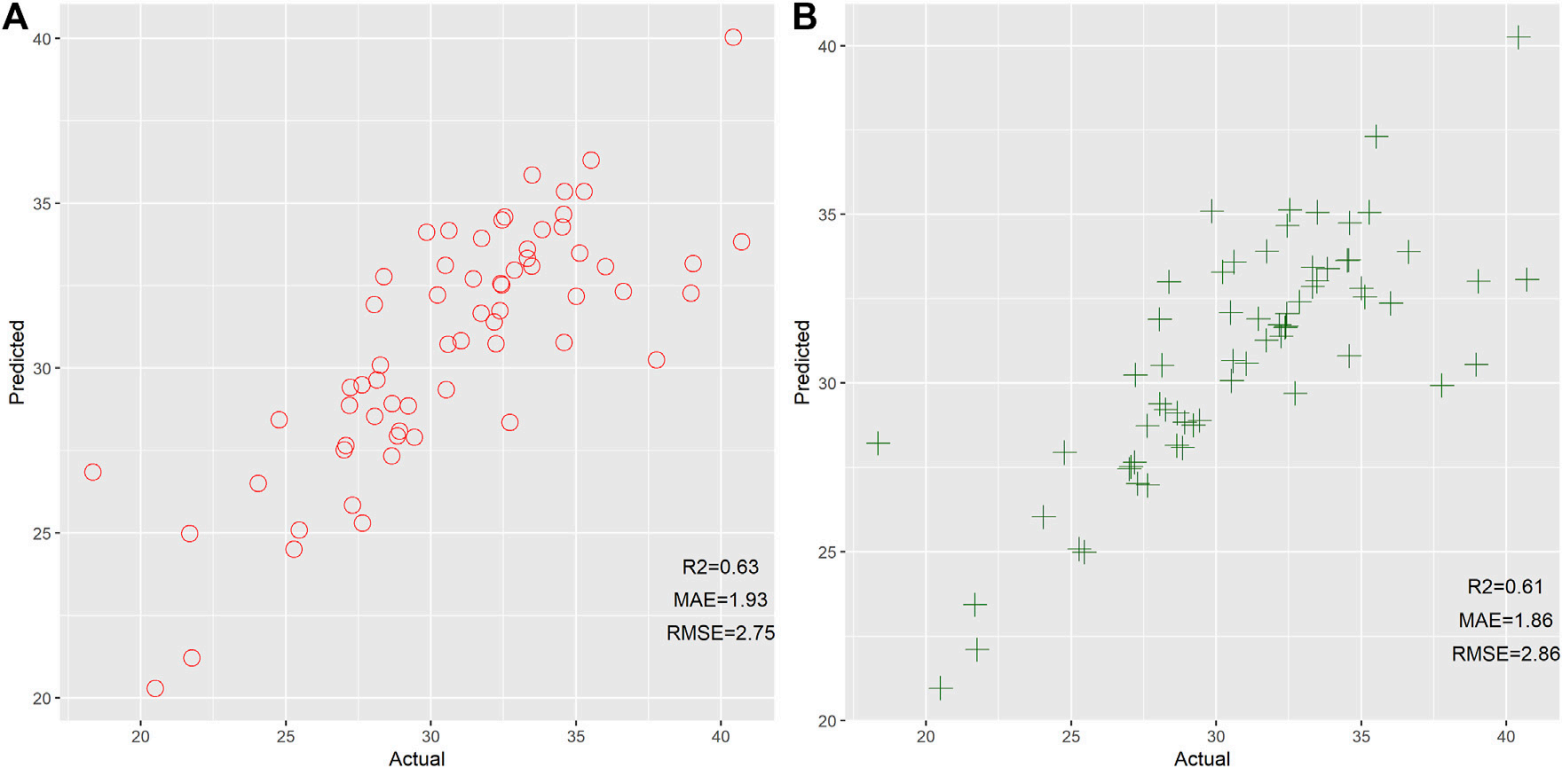
Scatter plot of the predicted age and actual age by the generalized linear model **(A)** and support vector machine **(B)** based on 14 mRNAs.

Next, we also assessed the efficiency of GLM and SVM for age estimation based on 14 mRNAs, as shown in Figure 3. Results reflected that GLM and SVM showed comparable performance for age prediction. Even so, we found that these two models developed by the GLM and SVM exhibited worse performance than the RF model. Therefore, we stated that RF could be viewed as the preferable machine learning method for age prediction in this study. In comparison to previous studies (Zubakov et al., 2016; Fang et al., 2020; Wang et al., 2022), relatively low MAE and RMSE between actual and predicted results were observed in the current study. We postulated that these results might be related to a small age bracket (18–41), which leads to low MAE and RMSE. Therefore, we need to collect more individuals with different age ranges to further evaluate the performance of these 14 mRNAs for age estimation.

It should be noted that there are some shortcomings in the current research. First, age-associated mRNAs were only selected based on peripheral blood samples of female individuals. However, the aging process showed gender- and tissue-specific changes (Gomez-Verjan et al., 2018). Previous research revealed higher predicted accuracy of age was observed in males than females based on the RNA markers (Zubakov et al., 2016; Fang et al., 2020; Wang et al., 2022). Given these findings, we stated that these 14 mRNAs could achieve better age estimation in male individuals, but the application values of these 14 mRNAs in males and other tissues need to be assessed in the future. Second, the study was conducted based on the previously reported data. We need to validate the expression level of these 14 mRNAs by real-time PCR. Third, the studied individuals are European individuals. We were not sure whether the obtained results were suitable for Chinese individuals, given the large genetic differentiation between Chinese and Europeans. Accordingly, expression levels of these 14 mRNAs in Chinese individuals with different age ranges remain to be further evaluated.

### Gene Set Enrichment Analysis of Genes Associated With Age

**FIGURE 4:**
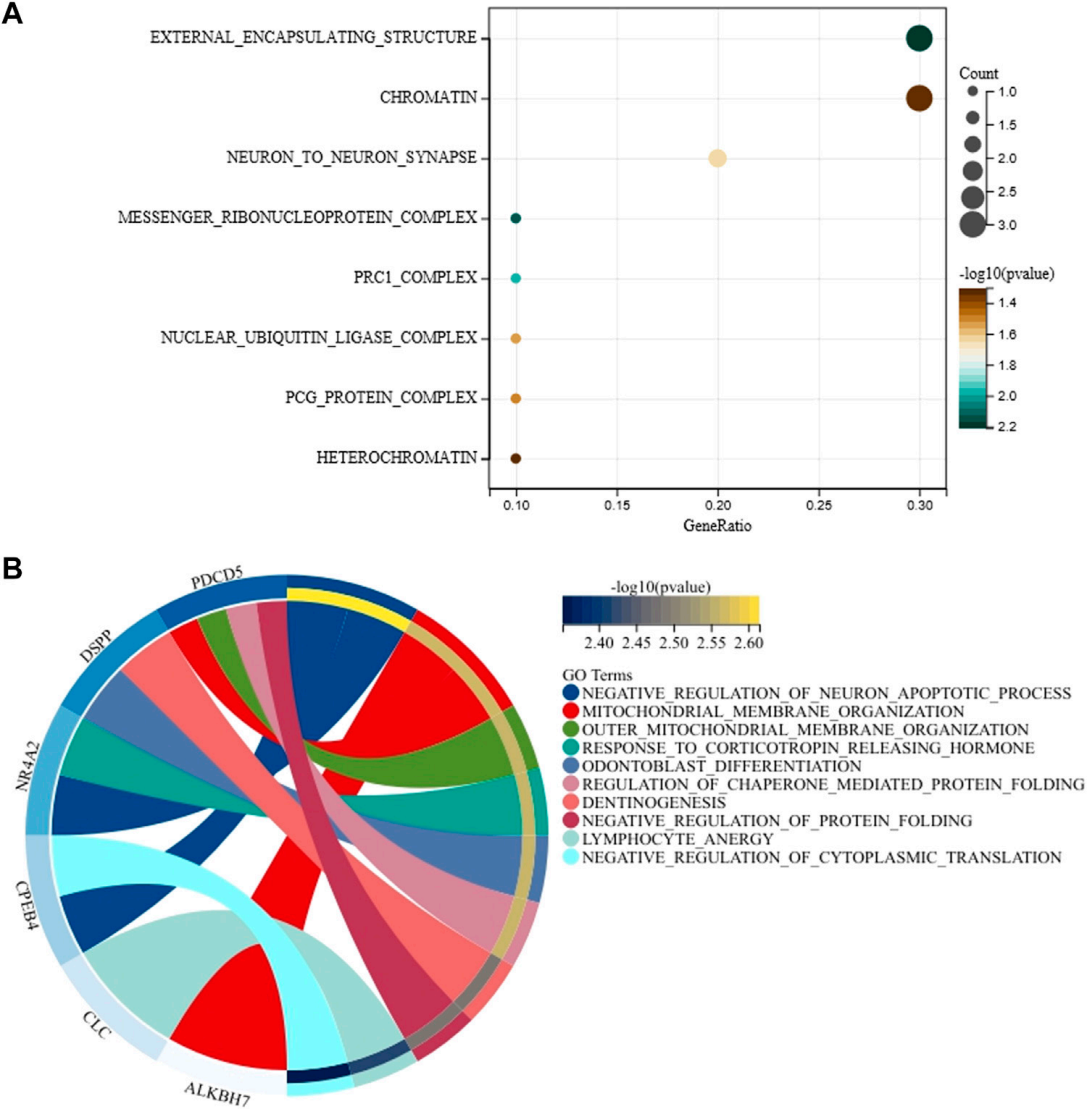
Gene ontology analysis for cellular components **(A)** and biological processes **(B)** of 14 genes associated with age.

Gene ontology analyses of 14 genes that correspond to 14 mRNA markers were conducted to explore the molecular function, biological process, and cellular components of these 14 genes. As shown in Figure 4A and Supplementary Table S6, we found that these 14 genes were related to external encapsulating structure, messenger ribonucleoprotein complex, prc1 complex, neuron to neuron synapse, nuclear ubiquitin ligase complex, pcg protein complex, chromatin, heterochromatin, etc. Even so, these cellular components did not show statistically significant relationships with 14 genes after Benjamini–Hochberg correction. For biological processes of these 14 genes, we found that the *PDCD5* gene is mainly related to outer mitochondrial membrane organization, mitochondrial membrane organization, regulation of chaperone-mediated protein folding, and negative regulation of protein folding; the *DSPP* gene is mainly related to odontoblast differentiation and dentinogenesis; the *NR4A2* gene is mainly involved in negative regulation of the neuron apoptotic process and response to corticotropin-releasing hormone; the *CPEB4* gene is mainly implicated in the negative regulation of the neuron apoptotic process and negative regulation of cytoplasmic translation; the *ALKBH7* gene is mainly related to mitochondrial membrane organization (Figure 4B and Supplementary Table S7). However, no statistically significant biological processes were observed for these 14 genes when Benjamini–Hochberg correction was applied. Molecular function and Kyoto Encyclopedia of Genes and Genomes (KEGG) pathway analysis of 14 genes are shown in Supplementary Figure S2 and Supplementary Tables S8, S9. Likewise, we did not observe statistically significant molecular function and KEGG pathway for these 14 genes after Benjamini–Hochberg correction.

## Conclusion

To sum up, 14 mRNAs related to age were identified from the genome-wide expression data, which showed relatively high correlations with aging. In addition, three machine learning methods were used to build models for age estimation based on selected 14 mRNA markers. We found that the RF showed the best performance in comparison to the two algorithms, which could be viewed as the preferable method to develop the model for age prediction. Anyway, expression patterns of selected 14 mRNAs in Chinese individuals with different age ranges and other common tissues need to be further assessed in the future.

## Data Availability

Publicly available datasets were analyzed in this study. These data can be found at: http://www.ncbi.nlm.nih.gov/geo/query/acc.cgi? acc%25¼GSE27272.

## References

[B1] de Magalhães J. P. Curado J. Church G. M. (2009). Meta-analysis of Age-Related Gene Expression Profiles Identifies Common Signatures of Aging. Bioinformatics 25, 875–881. 10.1093/bioinformatics/btp073 19189975 PMC2732303

[B2] Demanelis K. Tong L. Pierce B. L. (2021). Genetically Increased Telomere Length and Aging-Related Traits in the U.K. Biobank. Journals Gerontol. - Ser. A Biol. Sci. Med. Sci. 76, 15–22. 10.1093/GERONA/GLZ240 PMC7756694 31603979

[B3] Deng X.-D. Gao Q. Zhang W. Zhang B. Ma Y. Zhang L.-X. (2017). The Age-Related Expression Decline of ERCC1 and XPF for Forensic Age Estimation: A Preliminary Study. J. Forensic Leg. Med. 49, 15–19. 10.1016/j.jflm.2017.05.005 28486142

[B4] Fang C. Liu X. Zhao J. Xie B. Qian J. liu W. (2020). Age Estimation Using Bloodstain miRNAs Based on Massive Parallel Sequencing and Machine Learning: A Pilot Study. Forensic Sci. Int. Genet. 47, 102300 10.1016/j.fsigen.2020.102300 32353697

[B5] Feng L. Peng F. Li S. Jiang L. Sun H. Ji A. (2018). Systematic Feature Selection Improves Accuracy of Methylation-Based Forensic Age Estimation in Han Chinese Males. Forensic Sci. Int. Genet. 35, 38–45. 10.1016/j.fsigen.2018.03.009 29631189

[B6] Ghatak A. (2017). Machine Learning with R, Birmingham UK; Packt Publishing, 396, 1–210. 10.1007/978-981-10-6808-9

[B7] Gok E. Fedakar R. Kafa I. M. (2020). Usability of Dental Pulp Visibility and Tooth Coronal Index in Digital Panoramic Radiography in Age Estimation in the Forensic Medicine. Int. J. Leg. Med. 134, 381–392. 10.1007/s00414-019-02188-w 31720771

[B8] Gomez-Verjan J. C. Vazquez-Martinez E. R. Rivero-Segura N. A. Medina-Campos R. H. (2018). The RNA World of Human Ageing. Hum. Genet. 137, 865–879. 10.1007/s00439-018-1955-3 30386939

[B9] Huan T. Chen G. Liu C. Bhattacharya A. Rong J. Chen B. H. (2018). Age-associated microRNA Expression in Human Peripheral Blood Is Associated with All-Cause Mortality and Age-Related Traits. Aging Cell 17, e12687. 10.1111/acel.12687 PMC5770777 29044988

[B10] Ibrahim S. F. Gaballah I. F. Rashed L. A. (2016). Age Estimation in Living Egyptians Using Signal Joint T-Cell Receptor Excision Circle Rearrangement. J. Forensic Sci. 61, 1107–1111. 10.1111/1556-4029.12988 27184828

[B11] Kennedy B. K. Berger S. L. Brunet A. Campisi J. Cuervo A. M. Epel E. S. (2014). Geroscience: Linking Aging to Chronic Disease. Cell 159, 709–713. 10.1016/j.cell.2014.10.039 25417146 PMC4852871

[B12] Kennedy S. R. Salk J. J. Schmitt M. W. Loeb L. A. (2013). Ultra-Sensitive Sequencing Reveals an Age-Related Increase in Somatic Mitochondrial Mutations that Are Inconsistent with Oxidative Damage. PLoS Genet. 9 (9), e1003794. 10.1371/journal.pgen.1003794 24086148 PMC3784509

[B13] Kotěrová A. Navega D. Štepanovský M. Buk Z. Brůžek J. Cunha E. (2018). Age Estimation of Adult Human Remains from Hip Bones Using Advanced Methods. Forensic Sci. Int. 287, 163–175. 10.1016/j.forsciint.2018.03.047 29674227

[B14] Liberzon A. Subramanian A. Pinchback R. Thorvaldsdóttir H. Tamayo P. Mesirov J. P. (2011). Molecular Signatures Database (MSigDB) 3.0. Bioinformatics 27, 1739–1740. 10.1093/bioinformatics/btr260 21546393 PMC3106198

[B15] Liu Y.-Y. Welch D. England R. Stacey J. Harbison S. (2020). Forensic STR Allele Extraction Using a Machine Learning Paradigm. Forensic Sci. Int. Genet. 44, 102194. 10.1016/j.fsigen.2019.102194 31698330

[B16] López-Otín C. Blasco M. A. Partridge L. Serrano M. Kroemer G. (2013). The Hallmarks of Aging. Cell 153, 1194–1217. 10.1016/j.cell.2013.05.039 23746838 PMC3836174

[B17] Mamoshina P. Kochetov K. Putin E. Cortese F. Aliper A. Lee W.-S. (2018). Population Specific Biomarkers of Human Aging: A Big Data Study Using South Korean, Canadian, and Eastern European Patient Populations. Journals Gerontol. - Ser. A Biol. Sci. Med. Sci. 73, 1482–1490. 10.1093/gerona/gly005 PMC6175034 29340580

[B18] Meng H. Zhang M. Xiao B. Chen X. Yan J. Zhao Z. (2019). Forensic Age Estimation Based on the Pigmentation in the Costal Cartilage from Human Mortal Remains. Leg. Med. 40, 32–36. 10.1016/j.legalmed.2019.07.004 31326671

[B19] Peters M. J. Joehanes R. Pilling L. C., Schurmann C. Conneely K. N. Powell J., Johnson A. D (2015). The Transcriptional Landscape of Age in Human Peripheral Blood. Nat. Commun. 6, 8570. Available at: http://www.embase.com/search/results?subaction=viewrecord&from=export&id=L606532757%0A . 10.1038/ncomms9570 26490707 PMC4639797

[B20] Nakamura S. Kawai K. Takeshita Y. Honda M. Takamura T. Kaneko S. (2012). Identification of Blood Biomarkers of Aging by Transcript Profiling of Whole Blood. Biochem. Biophysical Res. Commun. 418, 313–318. 10.1016/j.bbrc.2012.01.018 22266314

[B21] Naue J. Hoefsloot H. C. J. Mook O. R. F. Rijlaarsdam-Hoekstra L. van der Zwalm M. C. H. Henneman P. (2017). Chronological Age Prediction Based on DNA Methylation: Massive Parallel Sequencing and Random Forest Regression. Forensic Sci. Int. Genet. 31, 19–28. 10.1016/j.fsigen.2017.07.015 28841467

[B22] Pan F. Chiu C.-H. Pulapura S. Mehan M. R. Nunez-Iglesias J. Zhang K. (2007). Gene Aging Nexus: A Web Database and Data Mining Platform for Microarray Data on Aging. Nucleic Acids Res. 35, D756–D759. 10.1093/nar/gkl798 17090592 PMC1669755

[B23] Peña-Solórzano C. A. Albrecht D. W. Bassed R. B. Burke M. D. Dimmock M. R. (2020). Findings from Machine Learning in Clinical Medical Imaging Applications - Lessons for Translation to the Forensic Setting. Forensic Sci. Int. 316, 110538. 10.1016/j.forsciint.2020.110538 33120319 PMC7568766

[B24] Santolaria C. (2021). Machine Learning in Medicine, Boca Raton FL USA; CRC Press, 312. 10.3390/mol2net-07-11828

[B25] Votavova H. Dostalova Merkerova M. Fejglova K. Vasikova A. Krejcik Z. Pastorkova A. (2011). Transcriptome Alterations in Maternal and Fetal Cells Induced by Tobacco Smoke. Placenta 32, 763–770. 10.1016/j.placenta.2011.06.022 21803418

[B26] Wang J. Wang C. Wei Y. Zhao Y. Wang C. Lu C. (2022). Circular RNA as a Potential Biomarker for Forensic Age Prediction. Front. Genet. 13. 10.3389/fgene.2022.825443 PMC8858837 35198010

[B27] Obermeyer Z. Emanuel E. J., (2016). Predicting the Future - Big Data, Machine Learning, and Clinical Medicine. N. Engl. J. Med. 375, 1216–1219. Available at: http://www.nejm.org/doi/10.1056/NEJMp1609300 . 27682033 10.1056/NEJMp1606181 PMC5070532

[B28] Zubakov D. Liu F. Kokmeijer I. Choi Y. van Meurs J. B. J. van IJcken W. F. J. (2016). Human Age Estimation from Blood Using mRNA, DNA Methylation, DNA Rearrangement, and Telomere Length. Forensic Sci. Int. Genet. 24, 33–43. 10.1016/j.fsigen.2016.05.014 27288716

