# Supplementary figures and images for: Systematic Selection of Age-Associated mRNA Markers and the Development of Predicted Models for Forensic Age Inference by Three Machine Learning Methods
Source: Front Genet. 2022 Jul 1;13:924408. doi: 10.3389/fgene.2022.924408 (PMC9283997; doi:10.3389/fgene.2022.924408)

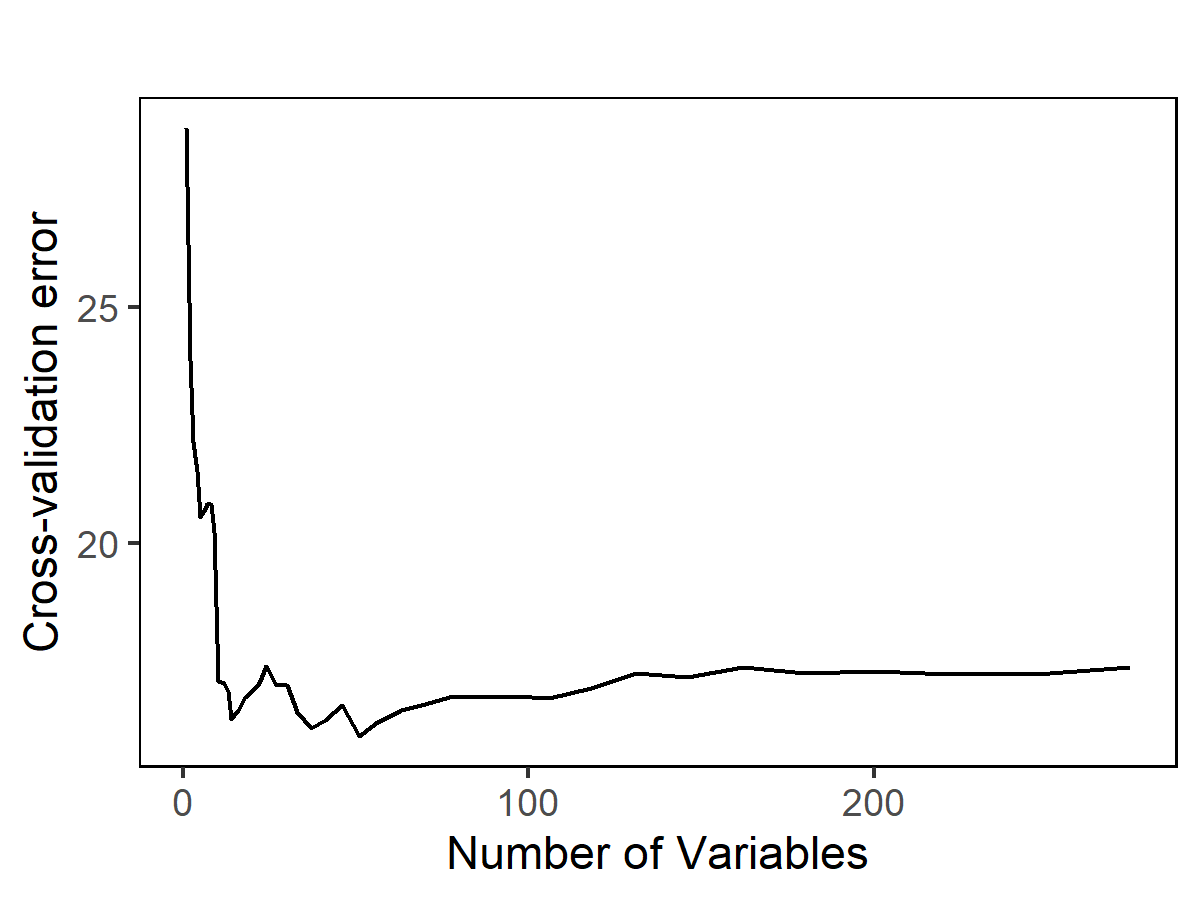

Supplement: Supplementary file 1 [file DataSheet1.ZIP › SM/Supplementary Figure 1.png]

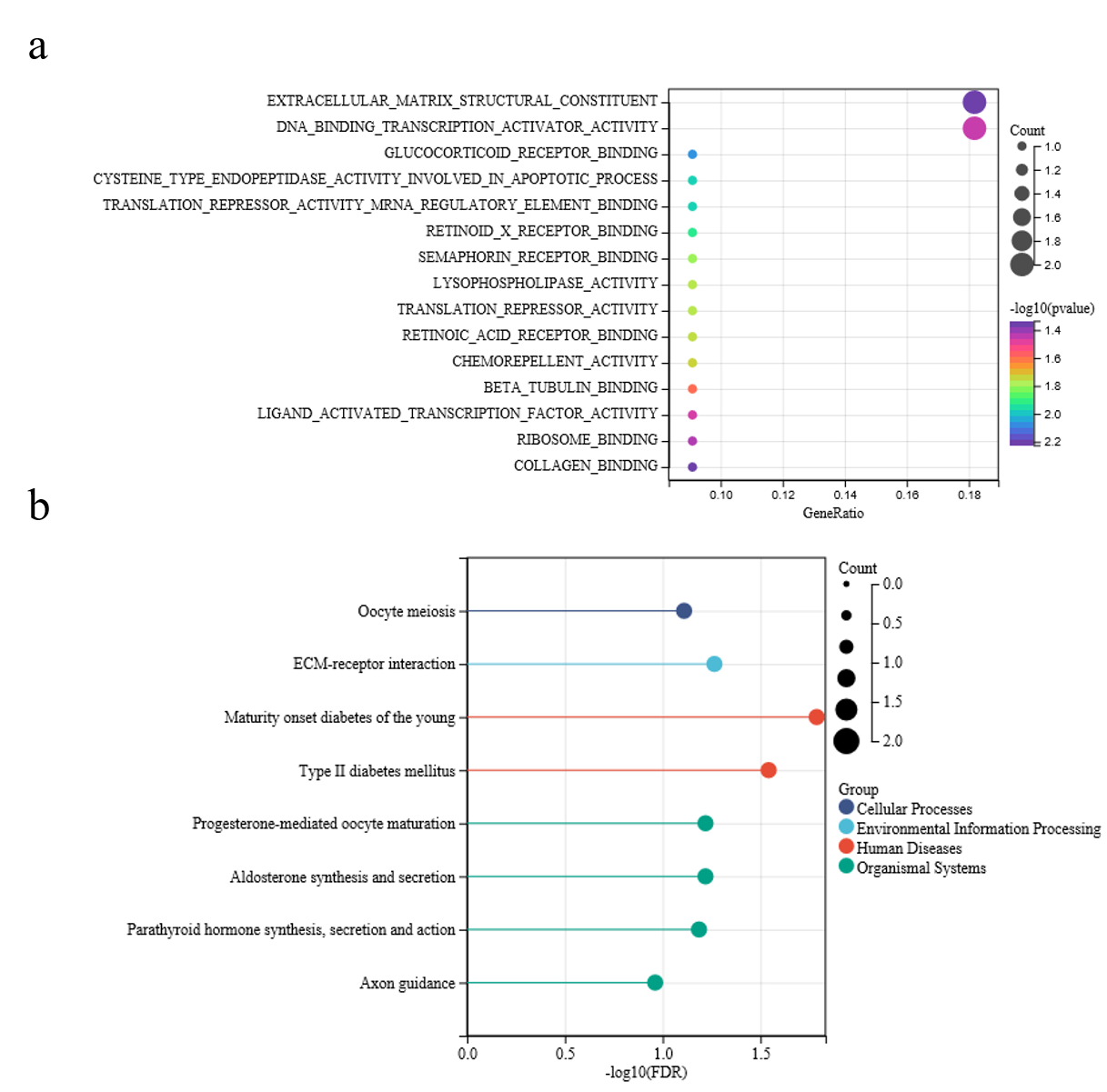

Supplement: Supplementary file 1 [file DataSheet1.ZIP › SM/Supplementary Figure 2.png]
